# Delayed Interval Delivery following Early Loss of the Leading Twin

**DOI:** 10.1155/2015/213852

**Published:** 2015-01-22

**Authors:** P. C. Udealor, I. V. Ezeome, F. C. Emegoakor, D. O. Okeke, P. C. N. Okere

**Affiliations:** ^1^Department of Obstetrics and Gynaecology, Niger Foundation Specialist Hospital (NFH), Enugu, Nigeria; ^2^Department of Radiology, NFH, Enugu, Nigeria

## Abstract

This was a case of a nulliparous woman with reduced chance of conception following unilateral salpingectomy and years of infertility. She eventually conceived following ovulation induction resulting in twin pregnancy. She had miscarriage that led to loss of one of the twins at 17 weeks of gestational age. The pregnancy was however continued for 116 days following meticulous management with eventual delivery of a live female baby with good outcome.

## 1. Introduction

Pregnancy loss before the age of viability is regarded as miscarriage and hardly anything can be done in some cases to stop the ongoing abortion process. In multifetal gestation, one of the fetuses can be lost and the remaining one/s retained. Reports have shown that such pregnancy can be continued with good outcome when properly managed; though, in some cases, serious maternal morbidities and even mortality can result. Delayed interval delivery became more necessary following increased cases of such pregnancy in women that conceived through assisted reproductive techniques and following years of infertility. It is obvious from the literature that there is no universally accepted protocol for managing such case, hence the need to add to the existing literature as the search for the best management protocol continues.

## 2. Case Report

The patient was Mrs. EC, a 31-year-old woman who was a banker by profession. She was G_2_P_0_^+1^. She first presented to us as a case of secondary infertility. Her previous pregnancy was ectopic for which she had laparotomy and salpingectomy.

At presentation, she was investigated and it was found that she had anovulatory infertility. She was placed on clomiphene citrate and became pregnant following the second course of the drug. Early scan done revealed twin pregnancy at 9 weeks of GA and she was registered for antenatal care. Her antenatal visits were unremarkable until GA of 17^+1^ weeks when she presented with drainage of liquor and subsequently expelled the leading twin. Umbilical cord was ligated high up in the cervix. Urgent scan done revealed that the remaining twin was viable with a separate placenta. Endocervical and high vaginal swabs were collected and sent for microscopy, culture, and sensitivity. She was placed on intravenous Augmentin (amoxycillin and clavulanic acid) and metronidazole for 3 days and then changed to oral forms for another 7 days and her vital signs were closely monitored. Vaginal swab microscopy, culture, and sensitivity were done at intervals and antibiotics given based on the result. Full blood count was equally done and was normal. She later opted to be discharged from the hospital. She was seen in the antenatal clinic every two weeks and ultrasound was done at each visit. Full blood count, prothrombin time, and fibrin degradation products estimation were equally done during the follow-up visits. At the gestational age of 30 weeks, the patient presented with bleeding per vaginam which was spontaneous. Scan done revealed viable fetus with EFW of 1.77 kg and showed that the bleeding was from the placenta of the expelled fetus. She was reassured and given dexamethasone injection for fetal lung maturation. The bleeding stopped after 2 days. Scan done at 32 weeks was reassuring and estimated fetal weight was 1.87 kg. At 34 weeks of GA, patient was seen in the clinic and there was no complaint. Scan done gave EFW of 2.287 kg with adequate liquor amnii. She was counseled on her condition but opted to continue with the pregnancy. Two days later at GA of 34^+2^, she presented to the emergency unit with complaint of drainage of liquor. Liquor drainage was confirmed and fetal heart rate was normal. She was counseled for emergency caesarean section which she consented to. She had the surgery and was delivered of a live female baby that weighed 2.5 kg with good Apgar score. Patient was seen in the postnatal clinic after 6 weeks and both mother and baby were normal.

## 3. Discussion

Delayed interval delivery in multiple pregnancies used to be a rare occurrence in obstetric practice. However with the emergence of different assisted reproductive techniques especially those that lead to multiple gestations, it has become a relatively common clinical condition.

Gestational age at delivery is an important factor in neonatal survival as this in most cases is directly related to fetal weight. Prolongation of gestational age and subsequent increase in fetal weight will lead to significant improvement in fetal outcome [1, 2]. This implies that the rate of survival after delayed delivery is higher compared to when delivery occurred at the time of initial delivery [3–6]. A delay of two or more days in the delivery of babies born before 30 weeks of gestation is associated with improved infant survival and higher infant birth weight [7]. Problems with early deliveries include increased duration of respiratory support, increase in the number of surgical procedures, and increased length of hospital stay. There is also increase in the frequency of necrotizing enterocolitis, patent ductus arteriosus, and retinopathy of prematurity [8].

The literature has quite some number of reports of cases of delayed interval deliveries but because of lack of consensus on the management protocol of those cases reported, there is still need for more reports on both the successfully and unsuccessfully managed cases. The case reported above had a delivery interval of 116 days with good outcome.

In all cases reported, there are generally agreed on criteria that should be met before undertaking delayed interval delivery. These include presence of multifetal gestation with delivery of the first fetus before the 30th week, diamniotic relationship between the presenting and subsequent fetus/fetuses, intact membranes in the remaining gestational sac, and also absence of fetal distress, lethal anomaly, abruption placenta, intra-amniotic infection, or maternal indications for delivery [1, 2, 6, 7, 9].

It is generally accepted that ligation of the umbilical cord after delivery should be high up in the cervix and with an absorbable suture. This helps to reduce the risk of infection [10].

The use of emergency cerclage in such patients is controversial. Some reports suggest that cerclage is associated with longer interdelivery interval [1, 11] while others found out that there was no statistically significant difference between those that had emergency cerclage and those that did not [12, 13]. They concluded that cerclage should be applied only when indicated. Cerclage was not used for our patient because there was no such indication.

In most of the previous reports, patients were on admission till the delivery of the remaining fetus/fetuses. They argued that such patient needs regular monitoring in the hospital because of the risk of chorioamnionitis. Some studies however reported successful outpatient management [11, 14]. Our patient though admitted for few weeks was later discharged because she was stable and always available when there was an appointment.

Tocolysis was an integral part of management in most of the documented reports. This is mainly because those patients presented with some form of contractions. Routine tocolysis is however debatable. Tocolysis is contraindicated in the presence of chorioamnionitis.

Antibiotics were used in all the reported cases. Broad spectrum antibiotics are preferred. The risk of chorioamnionitis is high and the sequelae may be disastrous for both the mother and the baby. Apart from preventing chorioamnionitis, antibiotics prolong latency interval which is a major indicator of outcome. Intravenous antibiotics are usually given for 3 days and oral ones are then given for another 7 days. Antibiotics use subsequently will depend on isolation of organisms from the cervicovaginal secretions.

Steroid use in delayed interval delivery is universally practiced because of the risk of preterm delivery of the remaining fetus. Some believe that delivery of subsequent fetus should be delayed till 28–32 weeks [15–18]. Another study concluded that continuation beyond 32 weeks is not necessary [19]. A study however delayed delivery till 36 weeks [20]. The author however believes that timing of delivery should be guided by the neonatal performance of the newborn unit of the institution and the perceived risk of infection. Our patient was delivered at the 34th week because there was no obvious sign of infection and the estimated fetal weight prior to then was low.

In most of the studies, patients were delivered through caesarean section but, in a study, the patient was delivered vaginally [21].

Ultrasonography is done routinely usually at every two weeks interval to assess the length and dilatation of the cervix and also to assess fetal growth and wellbeing. The findings may indicate the need for an earlier delivery. Full blood count, prothrombin time, and fibrin degradation product tests are done weekly to assess the patient and take further decisions regarding continuation or orderwise of the pregnancy. Cervicovaginal secretion microscopy, culture, and sensitivity test is also done weekly to detect any infection and treatment instituted early and appropriately. C-reactive protein estimation is also done as it is one of the early findings in infection. Suspicion of infection could also be raised on the basis of a rise in temperature and white blood count.

Despite reports of successfully managed cases, some complications can arise during and after management. These include chorioamnionitis that may lead to loss of the remaining fetus and sepsis for the mother. Reproductive career of the woman may be jeopardized. Life threatening complications like disseminated intravascular coagulopathy from retained placenta, abruption placenta, and septic shock can result from the procedure. Roman et al. described 31.6% incidence of serious maternal morbidity related to the procedure [22].

In conclusion, controversies still persist on how best to manage delayed interval delivery of remaining fetus/fetuses because such cases are still relatively rare. In multiple gestations achieved through assisted reproductive techniques, efforts should be made to prolong such pregnancy following spontaneous miscarriage or premature birth of one of the fetuses. More studies are needed to assess the long term outcome of such management for both the mother and her baby.
